# Intrinsic Genetic and Transcriptomic Patterns Reflect Tumor Immune Subtypes Facilitating Exploring Possible Combinatory Therapy

**DOI:** 10.3389/fmolb.2020.00053

**Published:** 2020-04-23

**Authors:** Yong Xu, Daixi Li, Zhenhao Liu, David L. Gibbs, Lu Xie, Guangrong Qin

**Affiliations:** ^1^Laboratory for Computational Biology, University of Shanghai for Science and Technology, Shanghai, China; ^2^Shanghai Center for Bioinformation Technology, Shanghai Academy of Science and Technology, Shanghai, China; ^3^Institute for Systems Biology, Seattle, WA, United States

**Keywords:** immune subtypes, breast invasive carcinoma, target, signaling pathways, combination therapy

## Abstract

The classification of immune subtypes was based on immune signatures highlighting the tumor immuno-microenvironment. It was found that immune subtypes associated with mutation and expression patterns in the tumor. How the intrinsic genetic and transcriptomic alterations contribute to the immune subtypes and how to select drug combinations from both targeted drugs and immune therapeutic drugs according to different immune subtypes are still not clear. Through statistical analysis of genetic alterations and transcriptional profiles of breast invasive carcinoma (BRCA) samples, we found significant differences in the number of somatic missense mutations and frameshift deletions among the different immune subtypes. The high mutation load for somatic missense mutations and frameshift deletions may be explained by the high frequency of mutations and high expression of DNA double-strand break repair pathway genes. Extensive analysis of signaling pathways in both the genetic and transcriptomic levels reveals significantly altered pathways such as tumor protein Tumor Protein P53 (TP53) and receptor tyrosine kinase (RTK)/RAS signaling pathways among different subtypes. Drug targets in the signaling pathways such as mitogen-activated protein kinase kinase kinase 1 (MAP3K1) and Phosphatidylinositol-4,5-Bisphosphate 3-Kinase Catalytic Subunit Alpha (PIK3CA) show genetic alteration in specific subtypes, which may be potential targets for patients of a specific subtype. More drug targets which show transcriptional difference among immune subtypes were discovered, such as cyclin-dependent kinase (CDK)4, CDK6, Erb-B2 receptor tyrosine kinase 2 (ERBB2), etc. Moreover, differences in functional activity between tumor growth and immune-related pathways also elucidate the extrinsic factors of differences in prognosis and suggest potential drug combinations for different immune subtypes. These results help to explain how intrinsic alterations are associated with the immune subtypes and provide clues for possible combination therapy for different immune subtypes.

## Introduction

In recent years, growing evidence has shown that immunosuppression in the tumor microenvironment (TME) is a major obstacle for effective antitumor therapy in patients (Munn and Bronte, 2016). The relationship between the infiltration level of immune cells in solid tumors and prognosis has been reported (Fridman et al., 2017). Solid tumors from diverse tissues of origin in The Cancer Genome Atlas (TCGA) have been classified into six immune subtypes, namely, wound healing (C1), interferon IFN-γ dominant (C2), inflammatory (C3), lymphocyte depleted (C4), immunologically quiet (C5), and transforming growth factor TGF-β dominant (C6) (Thorsson et al., 2018). The immune subtypes are associated with different prognoses and provide clues for immunotherapy response.

Some clinical successes are due to patient stratification, typically according to either the genetic features or immune environment, where it is hoped that more precise treatments can be delivered. Cancer immunotherapy demonstrates tremendous success in improving prognosis of some cancer types, including breast invasive carcinoma and melanomas (Li et al., 2016; Naik et al., 2019). Programmed cell death protein 1 (PD-1) and programmed death ligand 1 (PD-L1) antibodies have been shown to be effective in treating multiple malignancies (Gang et al., 2018); however, the drug efficacy depends on the mutational load (Hugo et al., 2016). Breast invasive carcinoma is a widely investigated tumor type with targeted drugs for different genetic subtypes. For example, the Phosphatidylinositol-4,5-Bisphosphate 3-Kinase Catalytic Subunit Alpha (PIK3CA) inhibitor alpelisib was approved in 2019 by the US Food and Drug Administration (FDA) for the treatment of PIK3CA-mutated hormone receptor-positive advanced breast invasive carcinoma as it significantly increases the progression-free survival for patients (Turner et al., 2015); human epidermal growth factor (EGF) receptor 2 human epidermal growth factor receptor-2 (HER2) antibodies such as trastuzumab and lapatinib can be used to treat HER2-positive patients; talazoparib, a poly (ADP-ribose) polymerase (PARP) inhibitor was approved in 2018 for the treatment of patients with breast cancer gene (BRCA) mutations and HER2-negative advanced or metastatic breast invasive carcinoma.

Although tumor patients can be classified into different immune subtypes, the biological mechanism that drives the differences in the immune microenvironment is not fully understood. How alterations in tumor cells induce specific tumor immuno-microenvironments or are associated with each other is still not clear. With multiple drug options for both immune therapy and targeted therapy, the understanding of the associated genetic factors of immune subtypes may provide new clues for precise drug or drug combinations.

For over 10 years, the TCGA has profoundly illuminated the multiple omics landscape of human malignancy. Cell composition methods, such as CIBERSORT (Newman et al., 2015) and TIMER (Li et al., 2016), have been developed to characterize complex tissue cell compositions. Using the genomic and transcriptomic data derived from bulk tumor samples, both TME and tumor genetic features from cancer cells can be explored, which can help to understand the association of tumor genetic features with TMEs, as well as exploring new drug combinations for different tumor subtypes.

In this study, based on the tumor immune subtypes identified in literature (Thorsson et al., 2018), we explored the genetic and transcriptional features for different immune subtypes. Through the integrative analysis of gene mutations, DNA damage response, and oncogenic signaling, we find an association of these pathways with immune subtypes and identified targeted drugs which are associated with different immune subtypes in breast invasive carcinoma. We also analyzed the interactions between key immune-related altered pathways and tumor growth pathways to explain the significant differences in prognosis among different immune subtypes.

## Materials and Methods

### Data Acquisition

Multiple omics data including gene expression data normalized by RSEM from Illumina HiSeq RNASeq, DNA somatic mutation data, and clinical data were downloaded from UCSC Xena (2018)^1^. In this study, two solid tumor types were selected, with breast invasive carcinoma (BRCA) as the research subject and lung adenocarcinoma (LUAD) as the comparative analysis and verification.

### Mutation Signature Analysis

Different mutational processes generate unique combinations of mutation types, termed “Mutational Signatures,” which have been classified based on the analysis of somatic mutation spectrum (Alexandrov et al., 2013). Based on tumor somatic mutation data in the TCGA database, the weights of mutation signatures (Alexandrov et al., 2013) for each tumor sample were calculated using the R packages “deconstructSigs” and “maftools” (Mayakonda et al., 2018). Kruskal–Wallis test was performed to estimate the difference of mutation signature weights among immune subtypes, and significant mutation signatures (*P*-value < 0.05) were selected among the immune subtypes. For pairwise analysis of mutation signature weights between immune subtypes, Wilcoxon rank-sum test was used. Significant results (*P*-value < 0.05) were shown in the boxplot using the R package “ggpubr.”

### Prognosis Analysis of Immune Subtypes

Survival analysis of tumors and relapse-free survival were performed using the R packages of “survminer” and “survival.” According to the overall survival time and relapse-free survival time in TCGA clinical data, the survival rates of different immune subtypes were compared and the survival curves were drawn. Log-rank test was performed to compare the difference of survival distribution between immune subtypes, *P*-values smaller than 0.05 were considered as significant difference in the survival rate of immune subtypes.

### Gene Set Variation Analysis

To calculate single-sample gene set enrichment, we used the gene set variation analysis (GSVA) program (Hanzelmann et al., 2013) to derive the absolute enrichment scores of previous literature reported DNA damage repair (DDR) (Knijnenburg et al., 2018) gene signatures as follows: (1) Base Excision Repair (BER), (2) Nucleotide Excision Repair (NER; including TC-NER and GC-NER), (3) Mismatch Repair (MMR), (4) Fanconi Anemia (FA), (5) Homologous Recombination (HR), (6) Non-Homologous End Joining (NHEJ), (7) Direct Repair (DR), (8) Translesion Synthesis (TLS), (9) Damage Sensor, etc., and oncogenic signaling pathway (Sanchezvega et al., 2018) gene signatures as follows: (1) cell cycle, (2) Hippo signaling, (3) Myc signaling, (4) Notch signaling, (5) oxidative stress response/Nrf2, (6) phosphatidylinositol 3-kinase (PI3K) signaling, (7) receptor tyrosine kinase (RTK)/RAS/mitogen-activated protein (MAP) kinase signaling, (8) TGF-β signaling, (9) tumor protein (TP)53 signaling, (10) b-catenin/Wnt signaling, and (11) Erb-B2 receptor tyrosine kinase (ERBB) signaling. To make a more comprehensive analysis of the functional modules, we further evaluated the activity of Kyoto Encyclopedia of Genes and Genomes (KEGG) pathways (gene sets) (Minoru and Susumu, 2000) within immune subtypes using the single-sample GSVA (ssGSVA). This method quantifies gene set enrichment in individual samples rather than at the group level.

### Mutational Status for Oncogenic Signaling Pathways

The mutational status for each signaling pathway is defined through a binary classification: if any gene was mutated in this pathway in one sample, the mutational status of this pathway in this sample was considered as mutated (use 1 to represent mutated status). On the contrary, the mutation status is set to non-mutated (use 0 to represent the non-mutated status). Fisher’s exact test was performed on the number of mutated samples and non-mutated samples within immune subtypes for each signaling pathway to compare the level of mutation in different immune subtypes.

### Drug Target Selection

To derive mutant genes associated with immune subtypes in breast invasive carcinoma and explore potential drug targets for each subtype, we detected gene mutations associated with each immune subtype using Fisher’s exact test (*P*-value < 0.05). The high-frequency mutant genes (mutation frequency > 5%) associated with each immune subtype were used to find linked therapeutic drugs using OncoKB^2^ and Drugbank^3^. Further, we selected the target genes in 11 signaling pathways from Drugbank and then compared whether the expression levels of these target genes were statistically significant among different immune subtypes using Wilcoxon rank-sum test. Target genes that are statistically significant (*P*-value < 0.05) were used to query DrugBank for drug selection.

### Correlation Analysis

The proliferation scores and leukocyte fractions for breast invasive carcinoma have been previously calculated (Thorsson et al., 2018). The enrichment scores of tumor growth and immune-related pathways in breast invasive carcinoma samples were estimated using GSVA on TCGA gene expression data. We measured the correlation coefficient between the proliferation scores and the enrichment scores of tumor growth-related pathways using Spearman’s rank correlation. Similarly, correlations between the enrichment scores of immune-related pathways and the leukocyte fraction were assessed using Spearman’s rank correlation. Significant correlations were considered as those pairs with *P*-value less than 0.05.

## Results

### Mutation Types and Mutation Signatures Are Associated With Immune Subtypes

**FIGURE 1:**
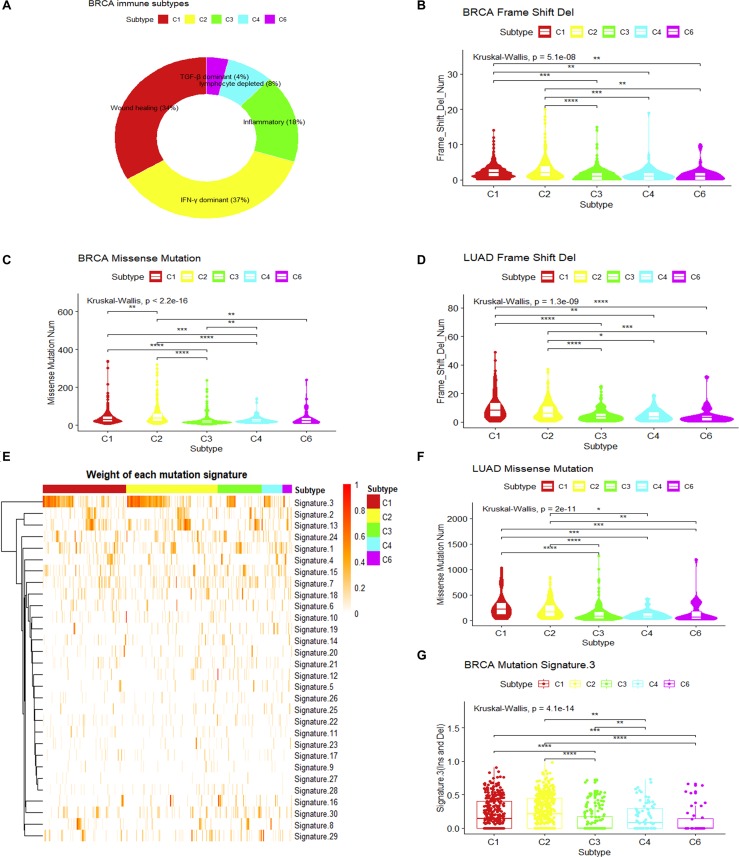
Mutation loads and mutation signatures in immune subtypes. **(A)** Sample proportions of different immune subtypes in breast invasive carcinoma. **(B,C)** Comparison of the frequency of frameshift deletion or missense mutation among different immune subtypes of breast invasive carcinoma (Wilcoxon rank-sum test was used. **P < 0.01, ***P < 0.001, ****P < 0.0001). **(D,F)** Comparison in the frequency of frameshift deletion or missense mutation among different immune subtypes of lung adenocarcinoma (LUAD) (Wilcoxon rank-sum test was used. *P < 0.05, **P < 0.01, ***P < 0.001, ****P < 0.0001). **(E)** The weight of each mutation signature of breast invasive carcinoma immune subtype. **(G)** The boxplot of the weight of mutation signature 3 for each immune subtype in breast invasive carcinoma (Wilcoxon rank-sum test was used. **P < 0.01, ***P < 0.001, ****P < 0.0001).

To understand the intrinsic tumor cell features that may drive the immune subtypes, we first asked whether there are differences in mutation types among immune subtypes. As mutated genes may produce altered neo-antigens, the mutation load and mutation types may have functional consequences for tumor cells and further drive the formation of immune microenvironments. Using breast invasive carcinoma in the TCGA dataset as an example, five immune subtypes can be detected according to the pan-cancer immune subtyping (Thorsson et al., 2018) (Figure 1A). Significant differences in the number of somatic missense mutations are found among different immune subtypes as well as significant difference in the number of somatic frameshift deletions (*P* < 10^–7^, Kruskal–Wallis test). The frequencies of frameshift deletion and missense mutations in the C1 and C2 immune subtypes were significantly higher than other subtypes (*P* < 0.01, Wilcoxon rank-sum test) (Figures 1B,C). In addition, consistent results were observed in LUAD (*P* < 0.05, Wilcoxon rank-sum test) (Figures 1D,F). This might hint that these types of mutations were important factors in generating the C1 and C2 immune subtypes in breast invasive carcinoma and LUAD. Both somatic missense mutation and frameshift deletion can introduce abnormal peptides, which may play a key role in recruiting immune cells.

Somatic mutations can be the consequence of multiple mutational processes, such as the deficiency in the DNA replication machinery and DNA repair system, abnormal enzymatic modification of DNA, or exposure to exogenous or endogenous mutagens (Alexandrov et al., 2013). From a large cohort of tumors, somatic mutation spectra have been categorized into 30 mutation signatures, which are associated with different biological processes (Alexandrov et al., 2013; Forbes et al., 2016). Using this concept, we measured the weight of different mutational signatures for each breast invasive carcinoma sample in TCGA and compared the difference of each mutation signature among immune subtypes (Alexandrov et al., 2013, 2015; Mayakonda et al., 2018). Results show that mutation signature 3 (MS3) showed significant differences among immune subtypes (*P*.adjust < 0.05, Kruskal–Wallis test, Benjamini and Hochberg adjustment) (Figure 1E). It is associated with the failure of DNA double-strand break repair by HR (Alexandrov et al., 2013; Forbes et al., 2016). The higher MS3 weights in C1 and C2 immune subtypes (*P* < 0.05, Wilcoxon rank-sum test) (Figure 1G) and the higher mutational load for somatic mutations and frameshift indels suggest that the formation of these two immune subtypes may result from the failure of a DNA double-strand break repair.

### Genetic Alteration and Expression Levels of DNA Damage Repair Shape the Immune Subtypes

**FIGURE 2:**
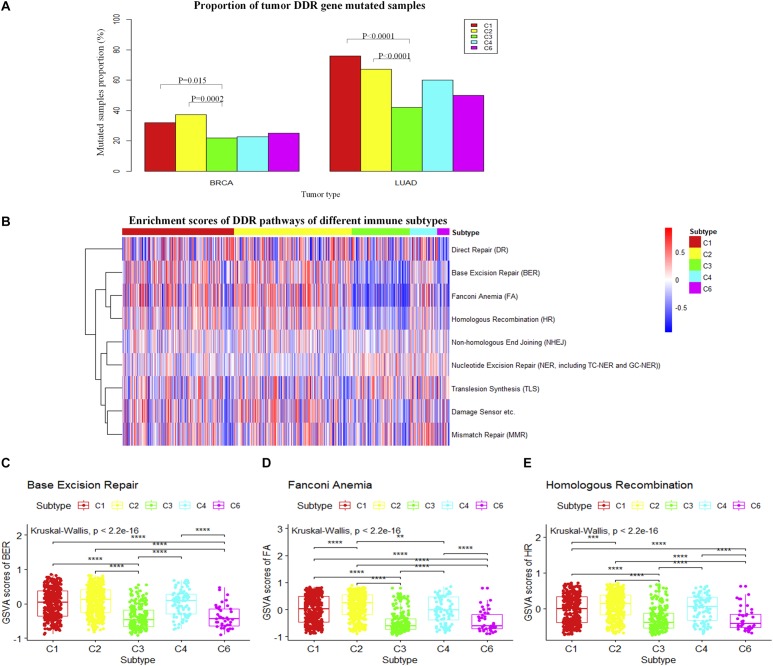
Relationship between immune subtypes and DNA damage repair (DDR) in breast invasive carcinoma. **(A)** The histogram shows the proportion of DDR gene mutations in different immune subtypes. **(B)** The heatmap shows the single-sample gene set variation analysis (ssGSVA) enrichment scores for DDR pathways from immune subtypes. **(C–E)** The boxplot shows the enrichment scores for breast invasive carcinoma immune subtypes in three DDR pathways (Wilcoxon rank-sum test was used. *P < 0.05, **P < 0.01, ***P < 0.001, ****P < 0.0001).

We then ask whether differences in the DDR system exist among immune subtypes. Loss of DDR function is an important determinant of cancer risk, progression, and therapeutic response (Jeggo et al., 2015). The proportions of samples with mutated DDR genes are shown in Figure 2A. The result shows that the proportion of mutated DDR genes in C1 and C2 subtypes of breast invasive carcinoma is significantly higher compared to that in the C3 subtype and slightly higher than those in C4 and C6. Similarly, the result was also observed in LUAD. However, these results do not fully explain how the DDR system interacts with the immune microenvironment.

We further compared the expression level of DDR genes among immune subtypes. ssGSVA (Hanzelmann et al., 2013) was performed for DDR-related pathways in breast invasive carcinoma (Figure 2B). BER, FA, and HR genes show higher expression in C1 and C2 subtypes and lower expression in C3 and C6 subtypes (*P* < 0.01, Wilcoxon rank-sum test) (Figures 2C–E). This indicates that the C1 and C2 subtypes may be more active in DDR and suggest genomic instability in these subtypes. Additionally, a similar result was found in LUAD (*P* < 0.01, Wilcoxon rank-sum test) (Supplementary Figures S1C–E). A promising way forward might be to select optimal drugs targeting the DDR pathway based on specific types of DDR mutations (O’Connor, 2015). The recent approval of olaparib, a PARP inhibitor for treating tumors harboring BRCA1 or BRCA2 mutations, provides a good example. The association of DDR features in tumor cells and the immune subtypes may provide new clues for selecting drug combinations for cancer treatment.

### Subtype-Specific Alterations in Signaling Pathways Provide Opportunities for Targeted Therapy and Immune Therapy

Current breast invasive carcinoma drugs are mostly targeted to signaling or cell cycle-related pathways, such as HER2 antibodies, PI3K inhibitors. To bridge the gap between targeted therapy and immune subtypes, we further investigated how signaling pathways are associated with tumor immune subtypes. Oncogenic signaling pathways in the TCGA have been reported to represent the individual and co-occurring actionable alterations which also suggest opportunities for targeted and combination therapies (Sanchezvega et al., 2018). The reported oncogenic pathways as well as well-known drug targetable signaling pathways that include (1) cell cycle, (2) Hippo signaling, (3) Myc signaling, (4) Notch signaling, (5) oxidative stress response/Nrf2, (6) PI3K signaling, (7) RTK/RAS/MAP kinase signaling, (8) TGF-β signaling, (9) TP53 signaling pathway, (10) b-catenin/Wnt signaling, and (11) ERBB signaling were further analyzed to understand the association of signaling pathways and immune subtypes (Sanchezvega et al., 2018). Oncogenic genes in each pathway which show genetic alterations are shown in Supplementary Table S1 (Sanchezvega et al., 2018).

**FIGURE 3:**
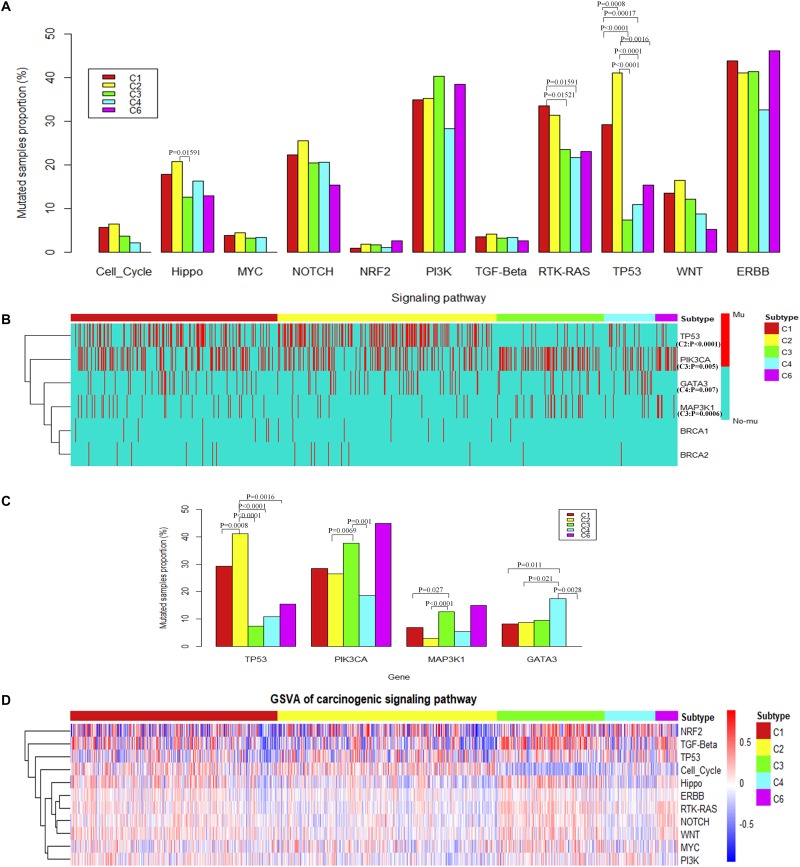
The alteration of carcinogenic signaling pathways in genetic mutations and transcriptional process. **(A)** Proportion of mutated samples for canonical signaling pathways in different immune subtypes (Fisher’s exact test, “Mutated samples proportion” is measured as the ratio of the number of samples with mutation in the pathway among the total number of samples in each immune subtype). **(B)** Genes with significant difference of mutations among different immune subtypes (Fisher’s exact test) or potential target genes for different immune subtypes. **(C)** The histogram shows the proportion of mutated samples for potential target genes in breast invasive carcinoma immune subtypes. **(D)** The heatmap shows enrichment scores of breast invasive carcinoma immune subtypes in canonical signaling pathways.

Results show that the alterations of genes in the TP53 signaling pathway were significantly overrepresented in C2 subtypes (Figure 3A). Specifically, the proportion of samples with TP53 mutations is significantly higher in the C2 subtype (Figure 3B). The alterations of genes in the RTK-RAS pathways were significantly overrepresented in the C1 subtype (Fisher’s exact test) (Figure 3A). Among these pathways, several potential target genes show a difference in mutation frequency among immune subtypes. PIK3CA and MAP kinase kinase kinase 1 (MAP3K1) were significantly high frequently mutated in C3 subtype, GATA3 was significantly high frequently mutated in C4 subtype (Fisher’s exact test) (Figures 3B,C), and BRCA1 or BRCA2 was mutated in a higher percentage of samples in C1 and C2, although not significantly different (Supplementary Figure S3A). PIK3CA is a key player in the ERBB signaling pathway which can be targeted by PI3K inhibitors (Wullenkord et al., 2019). So the immune subtype C3, which shows a higher mutation frequency in PIK3CA, may have a better response for PI3K inhibitors (Supplementary Figures S1A,B). MAP3K1 mutations are also reported to be associated with sensitivity to MAP kinase kinase (MEK) inhibitors in multiple cancer models (Zheng et al., 2018).

To consider the impact of molecular subtypes for our result, we performed enrichment analysis with the molecular subtypes in immune subtypes and the alteration of carcinogenic signaling pathways in molecular subtypes. Results show that HER2 subtype is associated with a significantly higher proportion of samples with mutations in the RTK–RAS signaling pathway (Supplementary Figure S2F). Meanwhile, HER2 subtype is significantly enriched in the C1 immune subtype (Supplementary Figure S2B), which is consistent with the significant mutation results of C1 subtype in the RTK–RAS signaling pathway (Figure 3A). We observed that HER2 subtype is also associated with a significantly higher proportion of samples with mutations in the ERBB signaling pathway; however, immune subtypes show a difference to that. Basal subtype is associated with a significantly higher proportion of samples with mutations in the p53 signaling pathway (Supplementary Figure S2F), while Basal is significantly enriched in the C2 immune subtype (Supplementary Figure S2A), which is consistent with the significant mutation results of C2 subtype in the TP53 signaling pathway (Figure 3A). TP53 gene is significantly mutated in Basal and HER2 subtypes (Supplementary Figure S2H), while Basal and HER2 subtypes are significantly enriched in C2 immune subtypes (Supplementary Figures S2A,B), which is consistent with the result that TP53 gene mutated significantly in C2 immune subtypes. PIK3CA and MAP3K1 gene is significantly mutated in Luminal A subtypes (Supplementary Figure S2H), while Luminal A subtypes are significantly enriched in C3 immune subtype (Supplementary Figure S2C), which is consistent with the results that PIK3CA and MAP3K1 gene mutated significantly in C3 immune subtypes. Previous study also suggested that PIK3CA and MAP3K1 alterations imply luminal A status in breast cancer and are associated with clinical benefits from PI3K inhibitors (Nixon et al., 2019). GATA3 gene is significantly mutated in Luminal B subtypes (Supplementary Figure S2H), while Luminal B subtypes are significantly enriched in C4 immune subtypes (Supplementary Figure S2D), which is consistent with the result that GATA3 gene mutated significantly in C4 immune subtypes. The enrichment analysis between the immune subtypes and the classical molecular subtypes suggest that, for different types of molecular subtypes, their immune environment also show different preference.

These results suggest that MAP3K1 and PIK3CA may be drug targets for patients in C3 subtype. GATA3 may be a potential therapeutic target for patients with the C4 subtype, and TP53 may be a potential therapeutic target for patients with the C2 subtype.

To further explore the differences among immune subtypes of breast invasive carcinoma in the transcriptomic level, from the perspective of signaling pathways, we also performed single-sample gene set enrichment analysis for the same 11 signaling pathways using breast invasive carcinoma samples (Figure 3D). Across immune subtypes, the 11 signaling pathways show statistical significance (*P*.adjust < 0.05, Kruskal–Wallis test, Benjamini and Hochberg adjustment) across immune subtypes. Since many drugs can target signaling pathways, understanding subtype-specific target gene expression will provide clues for drug selection. The target genes which show significant differences among immune subtypes are shown in Supplementary Figure S3B (*P* < 10^–6^, Kruskal–Wallis test). In total, our results showed 12 target genes, namely, ataxia telangiectasia mutated (ATM), B-Raf Proto-Oncogene, Serine/Threonine Kinase (BRAF), cyclin-dependent kinase (CDK)4, CDK6, EGF receptor (EGFR), ERBB2, fibroblast growth factor receptor (FGFR)1, FGFR2, neurotrophic receptor tyrosine kinase (NTRK)1, NTRK2, and protein kinase C alpha (PRKCA). The subtype-specific targets and drugs are shown in Table 1. Specifically, the high expression levels of CDK4 in C1 and C2 suggest the potential usage of CDK4 inhibitors such as palbociclib and related drugs. The high expression levels of ERBB2 in C3 suggest the potential usage of trastuzumab or lapatinib (or associated drugs). The association of signaling pathway alteration with expression and immune subtypes similarly may provide new ideas in combination drug therapy.

**TABLE 1:** Therapeutic drugs corresponding to target genes in signaling pathways and which immune subtypes are associated.

Target	Signaling pathways	Drug	Subtypes
ATM	TP53	Caffeine	C3, C6
BRAF	RTK-RAS, ERBB	Sorafenib	C3
		Vemurafenib	C3
		Regorafenib	C3
		Fostamatinib	C3
		Encorafenib	C3
		Dabrafenib	C3
CDK4	Cell Cycle	Palbociclib	C1, C2
		Abemaciclib	C1, C2
		Fostamatinib	C1, C2
		Ribociclib	C1, C2
CDK6	Cell Cycle	Palbociclib	C2, C6
		Abemaciclib	C2, C6
		Ribociclib	C2, C6
EGFR	RTK-RAS, ERBB	Cetuximab	C1, C2, C3, C6
		Gefitinib	C1,C2,C3,C6
		Erlotinib	C1,C2,C3,C6
		Lapatinib	C1,C2,C3,C6
		Lidocaine	C1,C2,C3,C6
		Necitumumab	C1,C2,C3,C6
		Zalutumumab	C1,C2,C3,C6
		Icotinib	C1,C2,C3,C6
		Vandetanib	C1,C2,C3,C6
		Afatinib	C1,C2,C3,C6
		Osimertinib	C1,C2,C3,C6
		Olmutinib	C1,C2,C3,C6
		Neratinib	C1,C2,C3,C6
		Brigatinib	C1,C2,C3,C6
		Dacomitinib	C1,C2,C3,C6
		Fostamatinib	C1,C2,C3,C6
		Panitumumab	C1,C2,C3,C6
		Zanubrutinib	C1,C2,C3,C6
ERBB2	RTK-RAS, ERBB	Afatinib	C3
		Brigatinib	C3
		Fostamatinib	C3
		Zanubrutinib	C3
		Lapatinib	C3
		Trastuzumab	C3
		Trastuzumab emtansine	C3
		Pertuzumab	C3
FGFR1	RTK-RAS	Regorafenib	C1,C3,C6
		Ponatinib	C1,C3,C6
		Sorafenib	C1,C3,C6
		Lenvatinib	C1,C3,C6
		Nintedanib	C1,C3,C6
		Fostamatinib	C1,C3,C6
		Erdafitinib	C1,C3,C6
FGFR2	RTK-RAS	Thalidomide	C3
		Regorafenib	C3
		Ponatinib	C3
		Nintedanib	C3
		Fostamatinib	C3
		Erdafitinib	C3
		Lenvatinib	C3
NTRK1	RTK-RAS	Imatinib	C3,C6
		Regorafenib	C3,C6
		Fostamatinib	C3,C6
		Larotrectinib	C3,C6
		Entrectinib	C3,C6
NTRK2	RTK-RAS	Larotrectinib	C3,C6
		Entrectinib	C3,C6
		Fostamatinib	C3,C6
NTRK3	RTK-RAS	Fostamatinib	C3,C6
		Larotrectinib	C3,C6
		Entrectinib	C3,C6
PRKCA	ERBB	Ellagic acid	C3,C6
		Midostaurin	C3,C6

*ATM, ataxia telangiectasia mutated; CDK, cyclin-dependent kinase; EGFR, epidermal growth factor receptor; ERBB, Erb-B2 receptor tyrosine kinase; FGFR, fibroblast growth factor receptor; NTRK, neurotrophic receptor tyrosine kinase; PRKCA, protein kinase C alpha; RTK, receptor tyrosine kinase; TP53, tumor protein 53. The mechanism of action is given as inhibitors or antagonists (Law et al., 2014).*

### Functional Behaviors in Immune Subtypes

**FIGURE 4:**
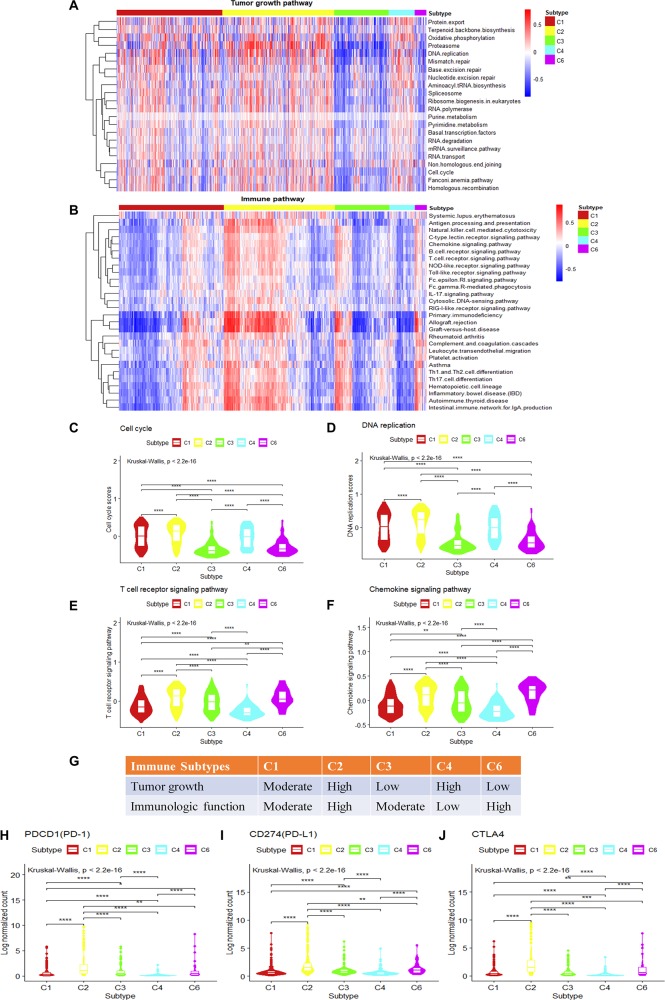
Single-sample gene set variation analysis (ssGSVA) of immune-related pathways and tumor growth-related pathways for different immune subtypes in breast invasive carcinoma. **(A,B)** The heatmap shows the ssGSVA enrichment scores of breast invasive carcinoma immune subtypes in tumor growth-related pathways and immune-related pathways. **(C,D)** The violin plot shows the ssGSVA enrichment scores of breast invasive carcinoma immune subtypes in cell cycle pathway and DNA replication pathway (Wilcoxon rank-sum test was used. ****P < 0.0001). **(E,F)** The violin plot shows the GSVA enrichment scores of breast invasive carcinoma immune subtypes in T cell receptor (TCR) signaling pathway and chemokine signaling pathway play (Wilcoxon rank-sum test was used. **P < 0.01, ****P < 0.0001). **(G)** Key characteristics of breast invasive carcinoma immune subtypes. **(H–J)** Differences in the expression levels of immune drug targets among breast invasive carcinoma immune subtypes. (Wilcoxon rank-sum test was used. *P < 0.05, **P < 0.01, ***P < 0.001, ****P < 0.0001).

The observation of cell growth potential and immune activities may help to explain prognosis and predict therapeutic opportunities in different subtypes. The tumor proliferation score represents the tumor growth activity, while the leukocyte fraction, to some degree, represents the level of immune activity. Although tumor proliferation and leukocyte fractions have been reported to be statistically significant in different immune subtypes (Wilcoxon rank-sum test) (Thorsson et al., 2018) (Supplementary Figures S4A,B), it is not known which functional gene modules cause the observed differences in tumor proliferation and immune microenvironment content. To make a more comprehensive analysis of the functional modules, we further expand our analysis from DNA damage processes and signaling pathways to a more comprehensive pathway set. Single sample gene set enrichment analysis was performed using KEGG pathways with the expression data of breast invasive carcinoma samples (Supplementary Figure S4C). The tumor growth-related pathways such as energy metabolism, transcription, translation, replication and repair, folding, sorting and degradation, cell growth and death, nucleotide metabolism show statistical significance among the gene set enrichment scores (*P*.adjust < 0.05, Kruskal–Wallis test, Benjamini and Hochberg adjustment) among different immune subtypes. The C2 and C4 subtypes present higher enrichment scores; on the contrary, the C3 and C6 subtypes show lower enrichment scores for the tumor growth-related gene sets (*P*.adjust < 0.05, Kruskal–Wallis test, Benjamini and Hochberg adjustment) (Figure 4A). It suggests that the C2 and C4 subtypes might be more active in tumor growth. Among these tumor growth-related pathways, cell cycle and DNA replication were positively correlated with tumor proliferation fraction (*P* < 0.05, Spearman correlation analysis) (Supplementary Figure S4D). Comparing gene expression in the cell cycle pathway or the DNA replication pathway among immune subtypes, the C2 and C4 subtypes show significantly higher enrichment scores in tumor growth-related pathways, followed by C1 subtype, C3 and C6 subtypes which have the lowest scores (Figures 4C,D). The low tumor growth enrichment scores for C3 and C6 subtypes indicate slow tumor growth.

We also performed GSVA using the immune-related pathways. Results show that 28 immune-related pathways were significantly different among immune subtypes (Figure 4B) (*P*.adjust < 0.05, Kruskal–Wallis test, Benjamini and Hochberg adjustment). Among these immune-related pathways, T cell receptor (TCR) signaling and chemokine signaling pathways were positively correlated with the leukocyte fraction (*P* < 0.05, Spearman correlation) (Supplementary Figure S4E). T cell development, differentiation, and maintenance are associated with the antigen-specific TCR and cytokine-mediated signals (Huang and August, 2015). Therefore, TCR signaling pathway and chemokine signaling pathway play a key role in regulating tumor immune microenvironment. The comparison of the gene expression in the T cell signaling pathways or chemokine signaling pathways among immune subtypes shows that the C2 and C6 subtypes have significantly higher enrichment scores, followed by C1 and C3 subtypes, and last, the C4 subtype (Figures 4E,F). This is consistent with the previous annotation for C4 as the lymphocyte-depleted subtype (Thorsson et al., 2018).

The C2 subtype shows a high expression level of tumor growth pathways as well as immune-related pathways, and the expressions of immune checkpoint genes such as PD-1, PD-L1, and cytotoxic T lymphocyte-associated antigen (CTLA)4 are also higher than other subtypes (Figures 4H–J). These results might suggest the potential usage of both anti-proliferation drugs and immune therapeutic drugs for this subtype. The C4 subtype is rapidly growing for tumor cells but without attracting much immune cells, which may result in a poor prognosis (Supplementary Figure S1A). It may also suggest that anti-proliferation drugs might work better for this subtype rather than immune therapy. The C6 subtype shows a high level of expression in immune-related pathways, but with low scores for tumor growth. The poor prognosis (Supplementary Figure S1A) for C6 might be caused by other factors that stimulate the activity of the immune system. As C6 is annotated as TGF-β dominant (C6), it also suggests a potential metastatic potential. The relatively high expression of immune drug targets (PD-L1, PD-1, CTLA4) in C6 also suggests the potential usage of immune therapy for this subtype. The C3 subtype shows the best prognosis, and with moderate levels of immune activity and slow tumor growth (Figure 4G), it may allow the potential drug combination for anti-proliferation drugs and immune therapeutic drugs. In conclusion, analysis of tumor growth and immune functional activity at the transcriptome level makes some progress in explaining the significant differences observed in the survival rates between immune subtypes as well as provides clues for drug combination selection.

## Discussion

The analysis of breast invasive carcinoma immune subtypes is hopefully beneficial to the diagnosis and treatment of breast invasive carcinoma. The pan-cancer classification of immune subtypes is based on immune-related gene sets and molecular markers previously reported in the literature (Thorsson et al., 2018). Our results suggest that mutation types, carcinogenic signaling pathways, and DDR machinery is associated with immune subtypes. The integrative analysis from tumor genetic features and immune subtypes may also provide clues for drug or drug combination selection.

In breast invasive carcinoma, two subtypes (C1 and C2) showed a high frequency of somatic missense mutations and frameshift deletions that may be a result of a failure of DNA double-strand break repair. This is supported by pathway enrichment analysis as well as the relatively high mutation frequency of BRCA1/BRCA2 in these two subtypes, suggesting the potential application of PARP inhibitors. Furthermore, C2 shows high expression of cell cycle-related drug targets such as CDK4, CDK6, as well as immune therapy-related drug targets such as PD-1, PD-L1, CTLA4, suggesting the potential combinatory usage of drugs from multiple categories.

The C3 subtype shows the best prognosis. In breast invasive carcinoma, this subtype shows a low mutation load (fewer number of somatic missense mutations and frameshift deletion) and is enriched with mutations in PIK3CA, with moderate immune activities and slow tumor growth. All these features suggest that the C3 subtype would be a candidate for treatment with drugs including PI3K inhibitors and anti-proliferation drugs. It also shows a potential for immune therapeutic drug response.

The C4 subtype in breast invasive carcinoma is found to have reduced immune activity coupled with active tumor growth indicating that the C4 subtype tumors are rapidly growing but without attracting immune cells, resulting in a poor prognosis (Supplementary Figure S1A). This subtype fits with the idea of “cold” tumors, which cannot be easily targeted by immune therapeutic drugs. So, anti-proliferation drugs might instead be considered.

The C6 subtype in breast invasive carcinoma shows high expression in immune-related pathways and low expression in the tumor growth-related pathways. The poor prognosis (Supplementary Figure S1A) for C6 might be caused by other factors that stimulate the activity of the immune system. C6 is annotated as TGF-β dominant (C6), increasing the potential for metastasis. High expression of PD-L1 and PD-1 in this subtype suggests the potential usage of immune therapy for this subtype.

The present study has several limitations. Although most of these observations are similar between breast invasive carcinoma and LUAD, there will likely be tissue-specific features. Therefore, there are likely additional factors that participate in immune subtype formation. Strong tissue specificity reflects differences in inflammatory or immune microenvironments of different tissues. The mutagenesis map of carcinogenic signaling suggests a certain relationship between the signaling pathways and the formation of immune subtypes. The degree to which mutations in signaling pathways are the driving force in the formation of the immune microenvironment still needs experimental verification. Furthermore, our results provide clues for finding drug combinations applicable to immune subtypes; however, for clinical practicality, more detailed experiments must be carried out.

## Conclusion

This study highlighted important factors potentially affecting the formation of immune subtypes in breast invasive carcinoma and elucidated the potential impact of canonical signaling pathways and DDR on immune subtypes. Functional activities from immune- and tumor growth-related pathways help explain the mechanisms by which there is a significant difference in patient survival between immune subtypes. This study also provides new clues for the therapeutic targets of immune subtypes of breast invasive carcinoma.

## Data Availability Statement

All datasets generated for this study are included in the article/Supplementary Material.

## Author Contributions

LX and GQ designed the research. YX conducted the research and wrote the manuscript. DL, ZL, DG, LX, and GQ revised the manuscript and performed some of the revised analyses. All authors read and approved the final manuscript.

## Conflict of Interest

The authors declare that the research was conducted in the absence of any commercial or financial relationships that could be construed as a potential conflict of interest.

## Footnotes

1 https://tcga.xenahubs.net/

2 https://www.oncokb.org/

3 https://www.drugbank.ca/

## Supplementary Material

FIGURE S1 Prognostic analysis of tumor immune subtypes and gene set variation analysis of DNA damage repair (DDR) in lung adenocarcinoma (LUAD) immune subtypes. **(A,B)** Overall survival analysis and recurrence-free survival analysis for all non-hematologic tumors. **(C–E)** The boxplot shows the gene set variation analysis (GSVA) enrichment scores of LUAD immune subtypes in three DDR pathways (Wilcoxon rank-sum test was used. **P* < 0.05, ***P* < 0.01, ****P* < 0.001, *****P* < 0.0001).

FIGURE S2 Enrichment analysis with the molecular subtypes in immune subtypes and the alteration of carcinogenic signaling pathways in molecular subtypes. **(A–E)** Enrichment analysis comparing BRCA molecular subtype distribution across C1–C6 immune subtypes (Fisher’s exact test was used. **P* < 0.05, ***P* < 0.01, ****P* < 0.001, *****P* < 0.0001). **(F)** Proportion of mutated samples for canonical signaling pathways in different molecular subtypes (Fisher’s exact test, “Mutated samples proportion” is measured as the ratio of the number of samples with mutation in the pathway among the total number of samples in each molecular subtype. **(G)** Genes with significant difference of mutations among different molecular subtypes or potential target genes for different molecular subtypes. **(H)** The histogram shows the proportion of mutated samples for potential target genes in breast invasive carcinoma molecular subtypes.

FIGURE S3 Mutation differences and expression differences of genes on signaling pathways in different immune subtypes in breast invasive carcinoma. **(A)** The histogram shows proportion of BRCA1 or BRCA2 mutated samples in breast invasive carcinoma immune subtypes. **(B)** Differences in the gene expression of known drug targets in the signaling pathways among breast invasive carcinoma immune subtypes. (Wilcoxon rank-sum test was used. **P* < 0.05, ***P* < 0.01, ****P* < 0.001, *****P* < 0.0001).

FIGURE S4 Pathways associated with tumor proliferation and immune microenvironment in immune subtypes. **(A,B)** Tumor proliferation and leukocyte fractions were statistically significant among different immune subtypes from breast invasive carcinoma (Wilcoxon rank-sum test was used. **P* < 0.05, ***P* < 0.01, ****P* < 0.001, *****P* < 0.0001). **(C)** The heatmap shows enrichment score of breast invasive carcinoma immune subtypes for KEGG pathways that cover a wide range of functionalities. **(D)** Bar plot of Spearman correlation ecoefficiency between the proliferation fraction and tumor growth related pathways enrichment scores in breast invasive carcinoma. **(E)** Bar plot of Spearman correlation ecoefficiency between the leukocyte fraction and immune-related pathways enrichment scores in breast invasive carcinoma.

TABLE S1 Gene set specification for oncogenic pathways used in the analysis.
