# Supplementary figures and images for: Intrinsic Genetic and Transcriptomic Patterns Reflect Tumor Immune Subtypes Facilitating Exploring Possible Combinatory Therapy
Source: Front Mol Biosci. 2020 Apr 23;7:53. doi: 10.3389/fmolb.2020.00053 (PMC7191006; doi:10.3389/fmolb.2020.00053)

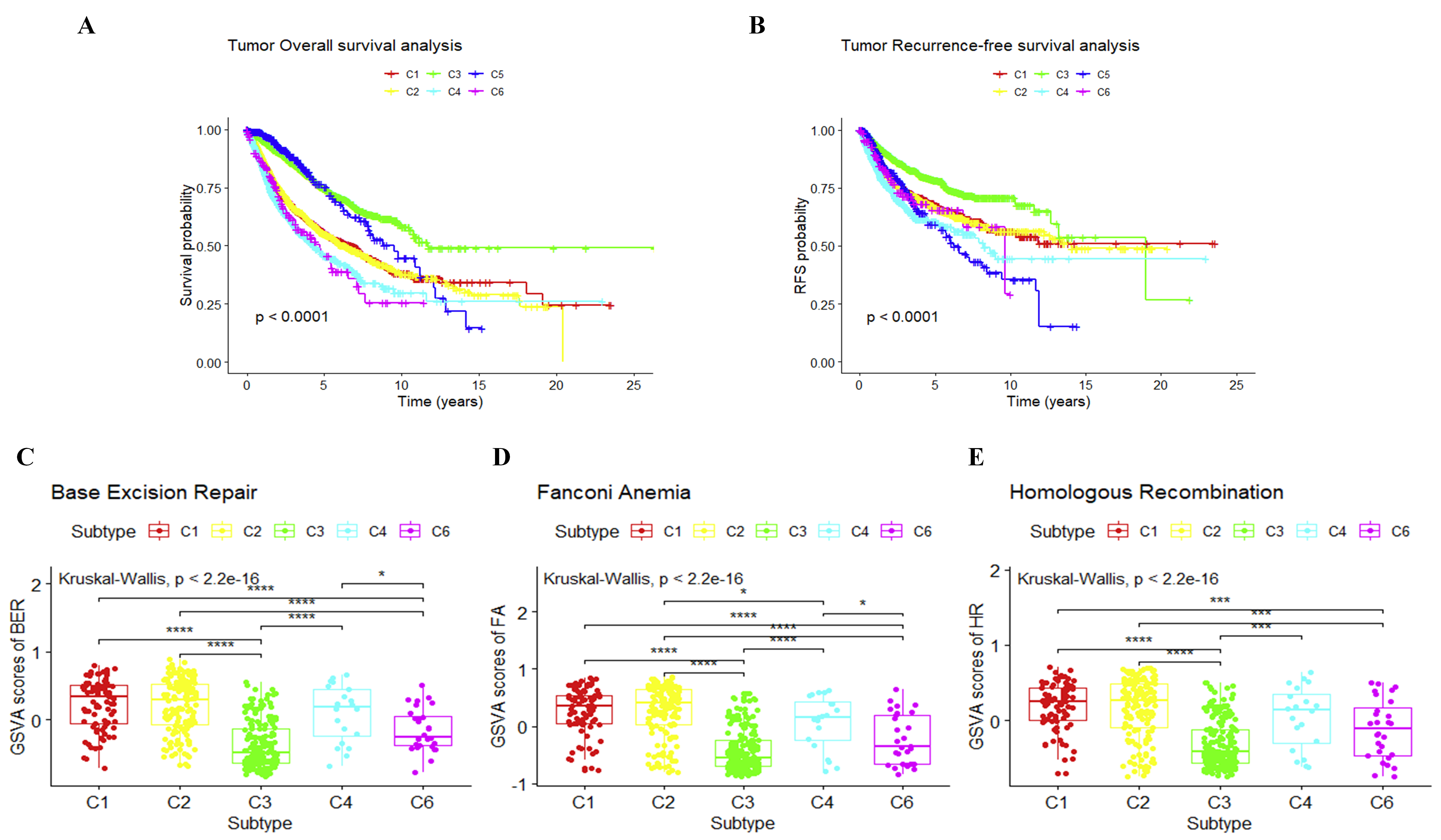

Supplement: FIGURE S1 — Prognostic analysis of tumor immune subtypes and gene set variation analysis of DNA damage repair (DDR) in lung adenocarcinoma (LUAD) immune subtypes. (A,B) Overall survival analysis and recurrence-free survival analysis for all non-hematologic tumors. (C–E) The boxplot shows the gene set variation analysis (GSVA) enrichment scores of LUAD immune subtypes in three DDR pathways (Wilcoxon rank-sum test was used. *P < 0.05, **P < 0.01, ***P < 0.001, ****P < 0.0001). [file Image_1.tif]

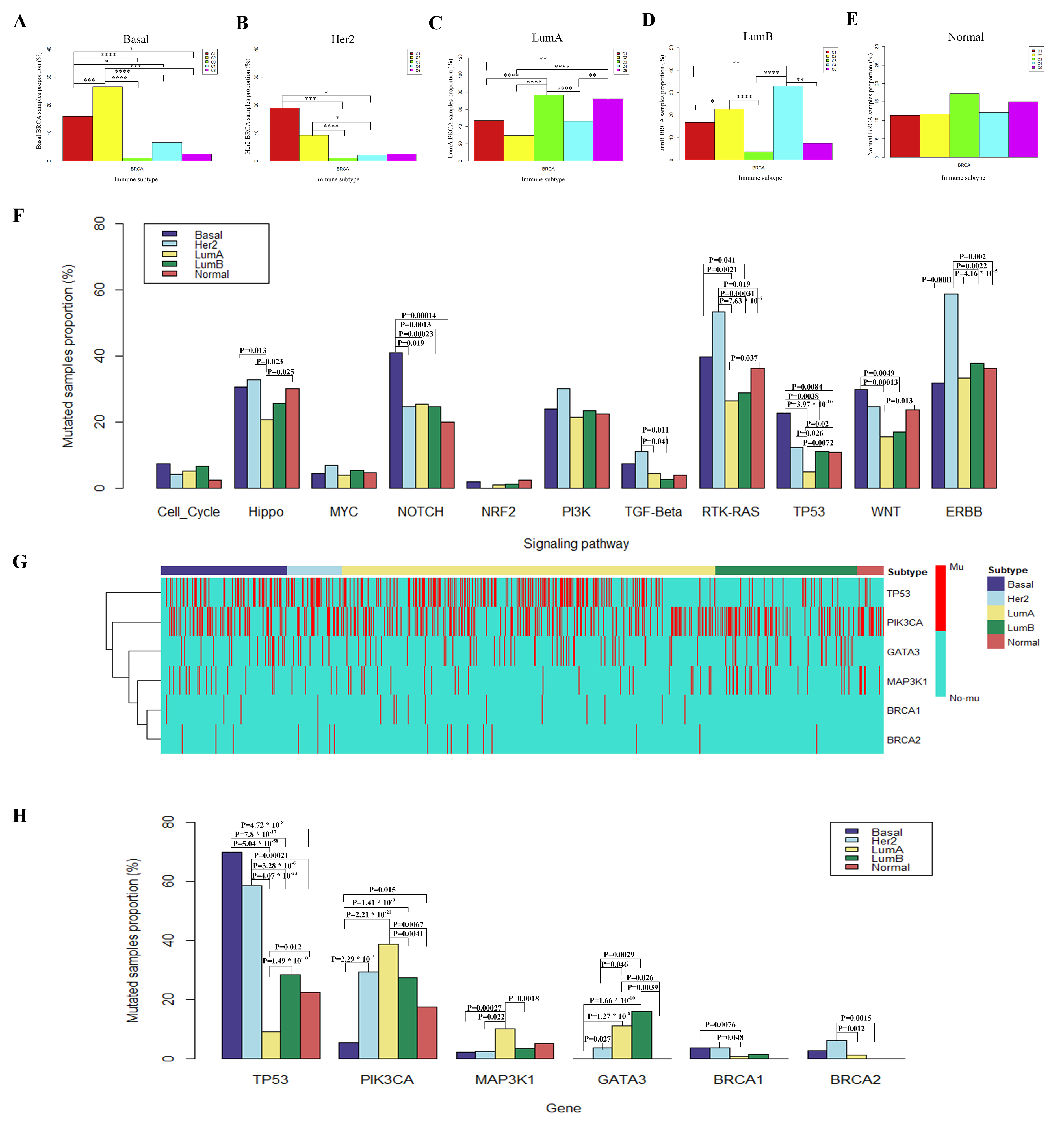

Supplement: FIGURE S2 — Enrichment analysis with the molecular subtypes in immune subtypes and the alteration of carcinogenic signaling pathways in molecular subtypes. (A–E) Enrichment analysis comparing BRCA molecular subtype distribution across C1–C6 immune subtypes (Fisher’s exact test was used. *P < 0.05, **P < 0.01, ***P < 0.001, ****P < 0.0001). (F) Proportion of mutated samples for canonical signaling pathways in different molecular subtypes (Fisher’s exact test, “Mutated samples proportion” is measured as the ratio of the number of samples with mutation in the pathway among the total number of samples in each molecular subtype. (G) Genes with significant difference of mutations among different molecular subtypes or potential target genes for different molecular subtypes. (H) The histogram shows the proportion of mutated samples for potential target genes in breast invasive carcinoma molecular subtypes. [file Image_2.TIF]

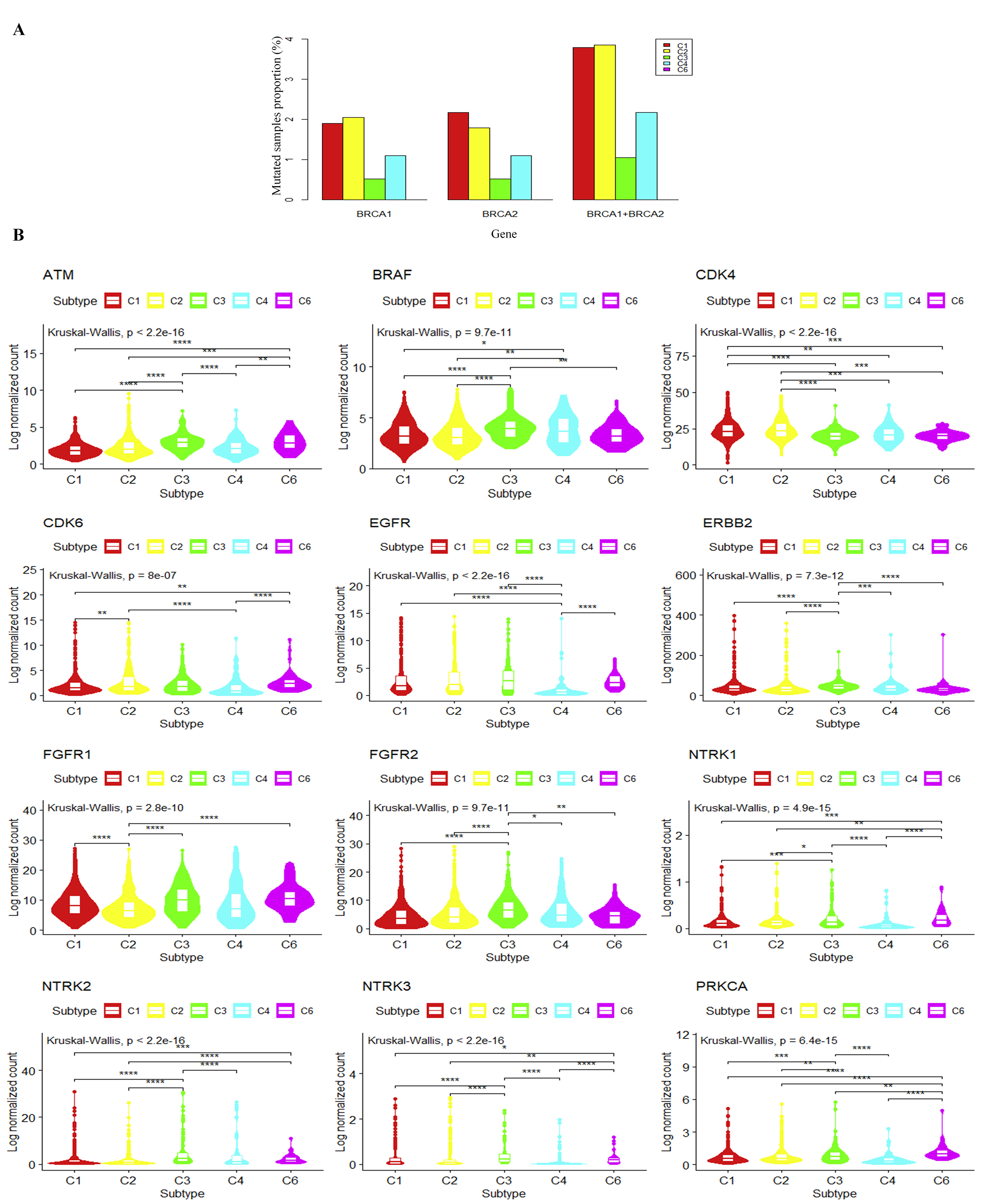

Supplement: FIGURE S3 — Mutation differences and expression differences of genes on signaling pathways in different immune subtypes in breast invasive carcinoma. (A) The histogram shows proportion of BRCA1 or BRCA2 mutated samples in breast invasive carcinoma immune subtypes. (B) Differences in the gene expression of known drug targets in the signaling pathways among breast invasive carcinoma immune subtypes. (Wilcoxon rank-sum test was used. *P < 0.05, **P < 0.01, ***P < 0.001, ****P < 0.0001). [file Image_3.TIF]

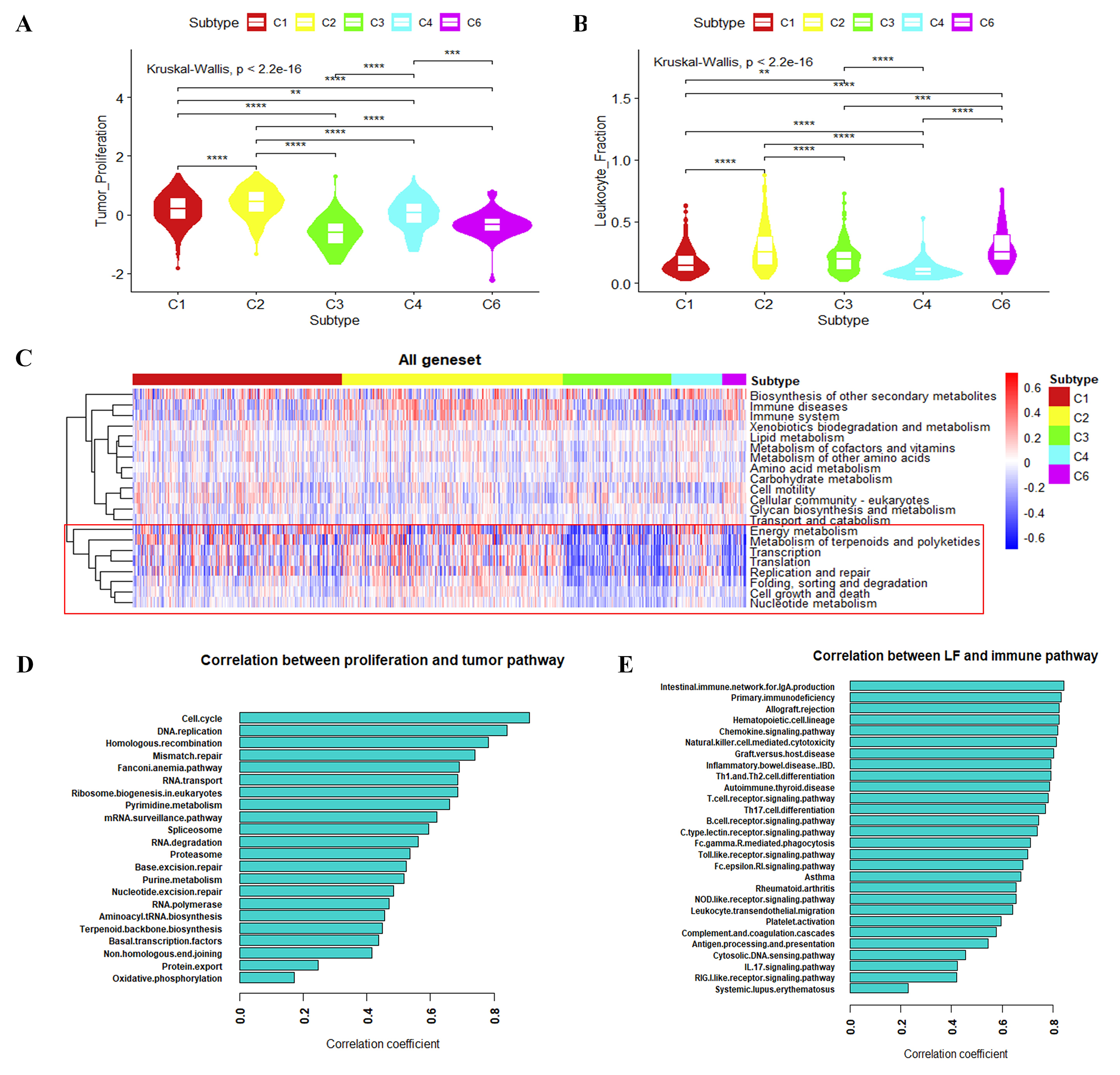

Supplement: FIGURE S4 — Pathways associated with tumor proliferation and immune microenvironment in immune subtypes. (A,B) Tumor proliferation and leukocyte fractions were statistically significant among different immune subtypes from breast invasive carcinoma (Wilcoxon rank-sum test was used. *P < 0.05, **P < 0.01, ***P < 0.001, ****P < 0.0001). (C) The heatmap shows enrichment score of breast invasive carcinoma immune subtypes for KEGG pathways that cover a wide range of functionalities. (D) Bar plot of Spearman correlation ecoefficiency between the proliferation fraction and tumor growth related pathways enrichment scores in breast invasive carcinoma. (E) Bar plot of Spearman correlation ecoefficiency between the leukocyte fraction and immune-related pathways enrichment scores in breast invasive carcinoma. [file Image_4.TIF]
